# Regulation of m6A Methylation as a New Therapeutic Option against COVID-19

**DOI:** 10.3390/ph14111135

**Published:** 2021-11-08

**Authors:** Carla Zannella, Luca Rinaldi, Giovanni Boccia, Annalisa Chianese, Ferdinando Carlo Sasso, Francesco De Caro, Gianluigi Franci, Massimiliano Galdiero

**Affiliations:** 1 Department of Experimental Medicine, University of Campania “Luigi Vanvitelli”, 80138 Naples, Italy; carla.zannella@unicampania.it (C.Z.); annalisa.chianese@unicampania.it (A.C.); massimiliano.galdiero@unicampania.it (M.G.); 2 Department of Advanced Medical and Surgical Sciences, University of Campania “Luigi Vanvitelli”, 80138 Naples, Italy; luca.rinaldi@unicampania.it (L.R.); ferdinando.sasso@unicampania.it (F.C.S.); 3 Department of Medicine, Surgery and Dentistry “Scuola Medica Salernitana”, University of Salerno, 84081 Baronissi, Italy; gboccia@unisa.it (G.B.); fdecaro@unisa.it (F.D.C.)

**Keywords:** SARS-CoV-2, COVID-19, epigenetics, methylation, m6A, rhein, fat mass and obesity-associated protein

## Abstract

The rapid spread of SARS-CoV-2 and the resulting pandemic has led to a spasmodic search for approaches able to limit the diffusion of the disease. The epigenetic machinery has aroused considerable interest in the last decades, and much evidence has demonstrated that this type of modification could regulate the early stages of viral infection. Recently it was reported that N6-methyladenosine (m6A) influences SARS-CoV-2 replication, although its role remains to be further investigated. The knockdown of enzymes involved in the m6A pathway could represent an optimal strategy to deepen the epigenetic mechanism. In the present study, we blocked the catalytic activity of the fat mass and obesity-associated protein (FTO) by using the selective inhibitor rhein. We observed a strong broad-spectrum reduction of infectivity caused by various coronaviruses, including SARS-CoV-2. This effect could be due to the modulation of m6A levels and could allow identification of this modification as a new therapeutic target to treat SARS-CoV-2 infection.

## 1. Introduction

Coronavirus disease 2019 (COVID-19) was first detected in China in 2019, developing into a pandemic after several months [1]. Despite the quick identification of the Severe Acute Respiratory Syndrome Coronavirus 2 (SARS-CoV-2) as the responsible pathogen in the novel infection, biological processes, pathological mechanisms, and its evolution are still debated [2]. To date, more than 4.7 million people around the world have died from COVID-19 [3]. Several therapeutic approaches have been proposed to treat both severe and mild cases of SARS-CoV-2 infection [4,5,6], but all of them have showed limited efficacy. A plethora of vaccines were developed and commercialized with record speed in terms of time to market [7,8]. Conjointly these approaches are limiting the infection spread but are still not enough to curb the pandemic. In this scenario, a deeper study of virus–host interaction will be the baseline to identify specific potential targets for therapeutic treatments of SARS-CoV-2. Among virus–host interactions, the epigenetic machinery plays a pivotal role [9]. Epigenetics includes all the changes that alter gene expression and cellular function but not the original nucleic acid sequence [10]. Although the exact function of these changes is unclear, they were reported to play a role in the viral life cycle [11,12,13,14]. In detail, it was already demonstrated that SARS-CoV-2 replication is partially controlled by epigenetic mechanisms [14,15,16,17]. Corley and Ndhlovu reported that methylation altered the angiotensin-converting enzyme 2 (ACE2) and interferon gene expression [18]. Recently N6-methyladenosine (m6A) modification was particularly investigated due to its functional role in SARS-CoV-2 replication and pathogenicity. It is a common and internal RNA modification altering both cellular and viral transcripts with important implications for RNA fate and function [19,20]. Three classes of enzymes are involved: (i) the writers, i.e., the methyltransferase complex consisting of methyltransferase-like 3 (METTL3), methyltransferase-like 14 (METTL14), Wilms’ tumor 1 (WT1)-associating protein (WTAP), methyltransferase-like 16 (METTL16), vir-like m6A methyltransferase associated (KIAA1429), and RNA binding motif protein 15 (RBM15) [21]; (ii) eraser enzymes, which include the demethylases fat mass and obesity-associated protein (FTO) and alkB homolog 5 (ALKBH5); and (iii) reader proteins, such as the YT521-B homology domain family (YTHDF) members, which are recruited m6A-binding proteins. SARS-CoV-2 infection influences not only the expression of these enzymes but also their localization. METTL3 levels increase after 48 h post-infection (hpi); meanwhile, the other methyltransferases, i.e., METTL14 and WTAP, are not altered. Expression levels of the eraser FTO are reduced 48 hpi, while ALKBH5 is not affected. All these enzymes are normally present in the nucleus but colocalize in the cytoplasm when the cell is infected [22]. Finally, YTHDF members, such as YTHDF1–3, YTH domain containing (YTHDC) 1, and YTHDC2, do not change during the infection [22]. Liu et al. demonstrated that an increase of viral replication occurred by knocking down METTL3, METTL14, and YTHDF2, while, on the contrary, when the ALKBH5 was knocked down, SARS-CoV-2 infection drastically decreased [23]. Additionally, other authors reported that depletion of METTL3 [22,24], YTHDF1, and YTHDF3 [24] affected SARS-CoV-2 and also the seasonal human β-coronavirus HCoV-OC43 replication by reducing viral RNAs and protein expression. Furthermore, Meng et al. indicated that RBM15 was highly expressed in severe COVID-19 patients, and its knockdown was associated with decreased cell death and inflammatory response [25]. Here, we asked whether selective inhibition of FTO catalytic activity using a small natural molecule, named rhein (4,5-dihydroxyanthraquinone-2-carboxylic acid), could restrict coronavirus replication. Rhein is an anthraquinone principally isolated from medicinal plants [26] and characterized by various biological activities, including the anti-inflammatory, antioxidant, and anticancer effects [27]. However, its role in m6A RNA regulation has been poorly analyzed during viral infection [28,29] even if, recently, Chen et al. suggested that rhein had a potent activity in inhibiting the angiotensin-converting enzyme 2 (ACE2) [30] by blocking the peptidyl dipeptidase activity. Therefore, the FTO eraser enzyme could be exploited as a novel potential therapeutic option by interfering with coronavirus replication and spread.

## 2. Results

### 2.1. Cytotoxicity Assay

**Figure 1:**
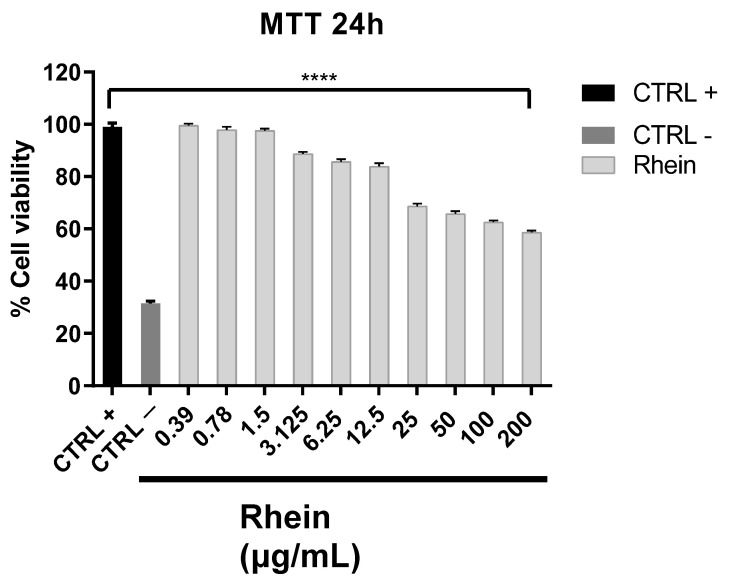
Cytotoxicity of rhein on Vero cells. Cells were treated with rhein and MTT assay was carried out after 24 h. The values are the mean ± SD of three independent experiments. The levels of statistical significance are indicated as follows: **** *p* < 0.0001. CTRL+: untreated cells; CTRL−: DMSO-treated cells.

To understand the potential rhein therapeutic window, we evaluated the cytotoxic effect via the MTT assay: Vero cells were treated with or without rhein (from 0.39 to 200 μg/mL) for 24 h. As reported in Figure 1, there was a reduction of 40% of cell viability at the highest tested concentrations.

### 2.2. Antiviral Activity

**Figure 2:**
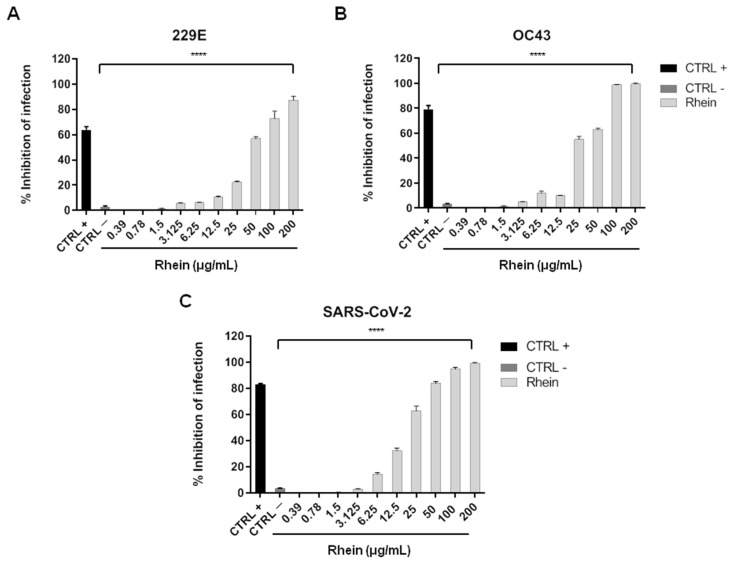
Antiviral activity of rhein against coronaviruses: (**A**) HCoV-229E; (**B**) HCoV-OC43; (**C**) SARS-CoV-2. Cells were exposed to virus and drug at the same time, and viral plaques were revealed after 48 hpi. The values are the mean ± SD of three independent experiments. The levels of statistical significance are indicated as follows: **** *p* < 0.0001. CTRL+: infected cells treated with 2 μg/mL ivermectin; CTRL−: infected cells.

To evaluate whether rhein could affect infection caused by coronaviruses, Vero cells were infected and simultaneously treated with the molecule at non-cytotoxic concentrations. Figure 2 shows the results obtained against three different human coronaviruses, which are the alphacoronavirus HCoV-229E and the two betacoronaviruses HCoV-OC43 and SARS-CoV-2.

Rhein caused a reduction in 229E replication starting from 50 μg/mL with a 60% inhibition of infection that increased up to 90% at 200 μg/mL (Figure 2A). Higher antiviral effects were registered against the betacoronaviruses. Starting from 25 μg/mL, rhein interfered with HCoV-OC43 (Figure 2B) and SARS-CoV-2 (Figure 2C) lifecycles and completely blocked the infection at 200 μg/mL. Interestingly, the active concentrations were not cytotoxic, highlighting the putative role of rhein in coronavirus inhibition.

### 2.3. RNA m6A Quantification

**Figure 3:**
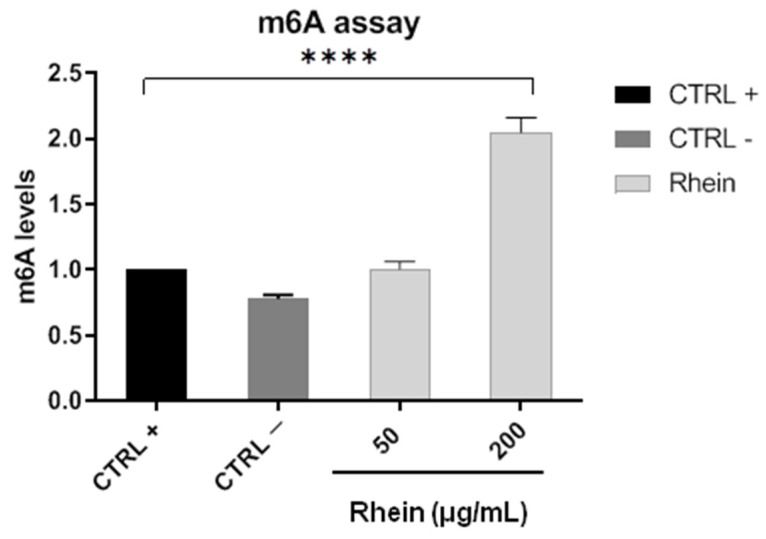
RNA m6A quantification. Cells were co-exposed to rhein and SARS-CoV-2, and total RNA was collected after 48 hpi. The values are the mean ± SD of three independent experiments. The levels of statistical significance are indicated as follows: **** *p* < 0.0001. CTRL+: not infected cells; CTRL−: infected cells.

Once it was demonstrated that rhein could inhibit viral replication by plaque assay, the next question was directed to investigate whether this biological observation could be ascribed to the modulation of m6A modification. Vero cells were mock-infected or infected with SARS-CoV-2 (MOI 0.01), and at 12 hpi, monolayers were treated with rhein (50 and 200 μg/mL) for 2 h. Then, RNA was extracted and methylation profiles evaluated in triplicate using the m6A RNA methylation assay kit (Figure 3). Methylation level increased in a dose-dependent manner. In detail, when the infected cells were treated with the highest concentration of rhein (200 μg/mL), a two-fold increase in the RNA m6A modification was observed. Following treatment with 50 μg/mL, rhein did not influence consistent methylation level compared to not-infected cells. This evidence could explain the total inhibition of SARS-CoV-2 replication observed through plaque assay when the inhibitor of FTO enzyme was used at the highest concentration.

## 3. Discussion

With the onset and rapid spread of COVID-19, the world is facing one of the biggest challenges in recent decades and, although vaccines offer a way out, other alternative strategies are still essential. The study of the epigenetic machinery stimulates considerable interest. In riboviruses, such as coronaviruses, epigenetic RNA modifications are very frequent. For instance, m6A methylation is an abundant and common hallmark that regulates different biological processes, including viral infection, affecting both cellular and viral transcripts. This modification has been widely reported in human immunodeficiency virus type 1 (HIV-1), identifying that m6A editing, and the recruitment of YTHDF proteins, stimulate HIV-1 mRNA stabilization and protein production [31]. The authors demonstrated that m6A caused a higher expression and recruiting of YTHDF proteins, in particular YTHDF2, resulting in greater HIV-1 replication. Conversely, in flaviviruses, such as hepatitis C virus (HCV) and Zika virus (ZIKV), m6A modification exerts a negative role in the viral replication, affecting neither the transcript stability nor the translation efficiency [32,33]. Gokhale et al. silenced the methyltransferases METTL3 and METTL14 (METTL3+14), observing a strong increase in the level of HCV NS5A protein; on the contrary, the FTO depletion caused a reduction in the viral protein production. Recently, with the advent of the current pandemic, many studies reported that the viral pattern of nonstructural proteins participating in the SARS-CoV-2 lifecycle could be regulated by histone deacetylases (HDACs) [14]. Furthermore, it is well known that the expression of ACE2 is finely controlled by DNA methylation and histone modifications [34,35,36]. In this scenario, a lot of effort is directed towards the use of epigenetic modulators as potential therapeutic options against SARS-CoV-2 infection [34,37,38]. However, how the m6A modification impacts SARS-CoV-2 replication is still controversial and has not been thoroughly investigated. Here, we showed that the replication of two common seasonal coronaviruses, i.e., HCoV-229E and OC43, and the pandemic SARS-CoV-2, could be inhibited by a small natural molecule named rhein, an inhibitor blocking the catalytic activity of the demethylase FTO. This protein plays an important role in a wide range of biological processes [39,40,41,42], even if it is mainly associated with obesity and adipogenesis [43,44]. To date, there is only one other study reporting the antiviral effect of rhein. In detail, Wang et al. described its anti-influenza A activity in the early stages of infection [45]. They reported a strong ability of rhein to inhibit the viral adsorption at 10 μg/mL. In our study, we reported an increased effect of rhein in interfering with betacoronaviruses replication (Figure 2) and, in particular, with SARS-CoV-2 infection. This effect was not ascribed to a potential toxicity of the molecule, as evidenced in a cytotoxicity assay performed on the same cellular model used for an antiviral assay (Figure 1). Probably, the strong anti-SARS-CoV-2 activity of rhein could be due to a regulation of the m6A pathway (Figure 3): when cells were infected and treated at the same time with the highest concentration of FTO inhibitor (200 μg/mL), there was a two-fold increase in the RNA m6A level. Our data is in accordance with what other studies previously reported: Liu et al. demonstrated that by silencing the other demethylase ALKBH5, SARS-CoV-2 infection drastically decreased [23].

## 4. Materials and Methods

### 4.1. Chemicals

Rhein (R7269) and Thiazolyl Blue Tetrazolium Bromide (M2128) were purchased from Sigma-Aldrich (St. Louis, MO, USA). The chemical structure of rhein is reported in Figure S1 (Supplementary Material). m6A RNA Methylation Assay Kit (ab185912) was acquired from Abcam (Cambridge, UK). All materials used for cell culture were purchased from HiMedia (Mumbai, India).

### 4.2. Cell Culture and Viruses

Vero cells (*Cercopithecus aethiops* epithelial kidney cells) were purchased by ATCC (Vero E6, CRL-1586) and grown in Dulbecco’s Modified Eagle’s Medium (DMEM) combined with 10% fetal bovine serum (FBS), 1 mM sodium pyruvate, 2 mM L-glutamine, and 1× penicillin and streptomycin. Human coronavirus strain HCoV-229E (ATCC VR-740), HCoV-OC43 (ATCC VR-1558), and SARS-CoV-2 (clinical isolate kindly donated by Hospital Lazzaro Spallanzani, Rome, Italy) were propagated on Vero cell monolayers as previously reported [46,47].

### 4.3. Cytotoxicity Assay

Vero cells were incubated overnight at 37 °C/5% CO_2_ in 96-well plate flat-bottom. Once cells reached confluence (2 × 10^4^ cells/well), rhein was added at different serial dilutions from 0.39 to 200 μg/mL and cytotoxicity was evaluated through MTT assay after 24 h of treatment as previously reported [48,49]. Data are expressed as the percentage of absorbance at 570 nm relative to the untreated cells (positive control, CTRL+).

### 4.4. Antiviral Assay: Co-Exposure

Vero cells were incubated overnight at 37 °C/5% CO_2_ in 12-well plate flat-bottom (3 × 10^5^ cells/well). The day after, co-exposure was performed as previously reported [48]: cells were treated with rhein at concentrations in the range 0.39–200 μg/mL and simultaneously infected with each virus at multiplicity of infection (MOI) of 0.01 for 2 h at 37 °C. Then, cell monolayer was covered with fresh culture medium supplemented with 5% carboxymethylcellulose (CMC) and incubated for 48 h. Vero cells were fixed, stained with crystal violet, and plaques were microscopically scored. The inhibition of viral infectivity was calculated as follows (1):

(1) $$\%\;\mathrm{viral}\;\mathrm{inhibition}=100-\frac{\begin{array}{c} (\mathrm{plaques}\;\mathrm{counted}\;\mathrm{in}\;\\ \mathrm{rhein}\;\mathrm{treated}\;\mathrm{cells}) \end{array}}{\begin{array}{c} (\mathrm{plaques}\;\mathrm{counted}\;\mathrm{in}\;\\ \mathrm{virus}\;\mathrm{treated}\;\mathrm{cells}) \end{array}}\times 100$$

### 4.5. m6A Analysis

Vero cells were infected with SARS-CoV-2 (MOI 0.01) for 12 h, then treated with rhein at 50 and 200 μg/mL. After 2 h of incubation, RNA was collected by TRI Reagent (T9424, Sigma-Aldrich) and quantified by NanoDrop ND-1000 UV-Vis (Thermo Scientific, Waltham, MA, USA). m6A levels were analyzed by m6A RNA methylation assay kit as already reported [49].

### 4.6. Statistical Analysis

Experimental data were elaborated using GraphPad Prism (version 6). Significant differences were analyzed by two-way analysis of variance (ANOVA) completed by Bonferroni post-test. Mean values were considered significantly different at *p* ≤ 0.05.

## 5. Conclusions

In summation, our results reveal that the inhibition of m6A demethylation and the simultaneous coronavirus infection negatively influences the viral replication. It provides proof of the concept that targeting FTO enzyme could lead to a new therapeutic option to treat SARS-CoV-2 infection.

## Data Availability

The data presented in this study are available on request from the corresponding author. Authors can confirm that all relevant data are included in the article.

## Supplementary Materials

The following are available online at https://www.mdpi.com/article/10.3390/ph14111135/s1, Figure S1: Chemical structure of rhein (C15H8O6).
