# Supplementary figures and images for: Regulation of m6A Methylation as a New Therapeutic Option against COVID-19
Source: Pharmaceuticals (Basel). 2021 Nov 8;14(11):1135. doi: 10.3390/ph14111135 (PMC8625908; doi:10.3390/ph14111135)

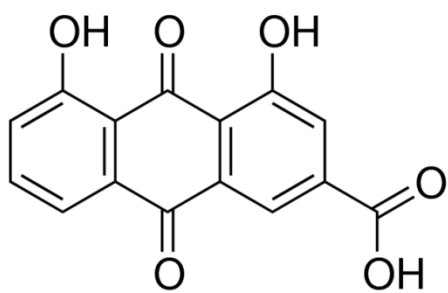

**Figure S1.** Chemical structure of rhein (C<sub>15</sub>H<sub>8</sub>O<sub>6</sub>).

Supplement: Supplementary file 1 [file pharmaceuticals-14-01135-s001.zip › pharmaceuticals-1423890-supplementary.pdf]
